# Effects of Cell Density and Microenvironment on Stem Cell Mitochondria Transfer among Human Adipose-Derived Stem Cells and HEK293 Tumorigenic Cells

**DOI:** 10.3390/ijms23042003

**Published:** 2022-02-11

**Authors:** Shalise A. Burch, Carlos Luna Lopez

**Affiliations:** Department of Biological Sciences, California State University San Marcos, 333 S. Twin Oaks Valley Rd., San Marcos, CA 92096, USA; shaliseanna@gmail.com

**Keywords:** adipose-derived stem cells, tumorigenic cells, HEK293, mitochondria, mitochondria transfer, microenvironment, cancer, tumor cells

## Abstract

Stem cells (SC) are largely known for their potential to restore damaged tissue through various known mechanisms. Among these mechanisms is their ability to transfer healthy mitochondria to injured cells to rescue them. This mitochondrial transfer plays a critical role in the healing process. To determine the optimal parameters for inducing mitochondrial transfer between cells, we assessed mitochondrial transfer as a function of seeding density and in two-dimensional (2D) and semi three-dimensional (2.5D) culture models. Since mitochondrial transfer can occur through direct contact or secretion, the 2.5D culture model utilizes collagen to provide cells with a more physiologically relevant extracellular matrix and offers a more realistic representation of cell attachment and movement. Results demonstrate the dependence of mitochondrial transfer on cell density and the distance between donor and recipient cell. Furthermore, the differences found between the transfer of mitochondria in 2D and 2.5D microenvironments suggest an optimal mode of mitochondria transport. Using these parameters, we explored the effects on mitochondrial transfer between SCs and tumorigenic cells. HEK293 (HEK) is an immortalized cell line derived from human embryonic kidney cells which grow rapidly and form tumors in culture. Consequently, HEKs have been deemed tumorigenic and are widely used in cancer research. We observed mitochondrial transfer from SCs to HEK cells at significantly higher transfer rates when compared to a SC–SC co-culture system. Interestingly, our results also revealed an increase in the migratory ability of HEK cells when cultured with SCs. As more researchers find co-localization of stem cells and tumors in the human body, these results could be used to better understand their biological relationship and lead to enhanced therapeutic applications.

## 1. Introduction

Adult stem cells (ASCs) are undifferentiated cells that can be found in almost every tissue type in the body [1]. ASCs are at the core of our internal repair system and have been largely studied for their capability of self-renewal and their potential to restore damaged tissues [1,2]. This has made them an ideal candidate for tissue engineering and regenerative medicine research [2,3,4]. It is thought that stem cells (SC) can differentiate to replace damaged cells; however, very few actually differentiate into functional somatic cells [5,6]. Current research suggests that ASCs are more likely to rescue cells through the transfer of RNA, organelles and paracrine effects [7,8,9].

Adipose-derived stem cells (ADSC) have been shown to be able to transfer their mitochondria [10,11,12] in order to mediate tissue repair [13,14,15,16]. Stem cells share their healthy mitochondria with injured cells in an attempt to rescue them [10,17,18]. Mitochondria transfer can occur through direct-contact nanotubes and gap junctions or by excretion and uptake of extracellular vesicles (EV) [7,10,11]. Mitochondria transfer plays a critical role in healing injured cells. By transferring mitochondria, SCs have the capability to restore oxidative phosphorylation [19,20] and replace dysfunctional mitochondria in a recipient cell [13,19]. By replacing damaged mitochondria, SCs are able to rescue the cell and protect it against further injury [10,14]. The ability of SCs to transfer mitochondria could lead to potential cures or treatments for mitochondrial disorders. However, if directed at tumorigenic cells, stem cell mitochondria transfer (SCMT) could enhance tumorigenesis. It has been shown that ADSC migrate selectively to tumors [7,13] and interact via direct contact [11,12,21] and paracrine mechanisms [6,15]. These interactions could enhance energy production [14,21] and trigger tumor formation [12,22]. In fact, after receiving mitochondria from SCs, tumor growth and migration have been reported to increase [23,24]. Thus, the use of SCs for tissue growth in certain cases (i.e., breast reconstruction [13]) could pose a high oncological risk.

We strived to better understand the dynamics of mitochondria transfer among ADSCs. We first sought to determine the optimal parameters for transfer between ADSCs. Cell culture parameters, such as seeding density, are very important when studying stem cell biology. They can affect cell growth, stem cell differentiation and apoptosis [25,26]. Additionally, controlling density allows for cells to be at varying distances from one another and plays a key role in whether a cell will have the ability to communicate with other cells via direct contact or through EVs [15,23,25]. Previous research has indicated that low seeding densities leads to increased EV production [25]. Similarly, a cell’s microenvironment and its ability to attach and move will ultimately affect its ability to communicate with nearby cells. Collagen type 1 is the most abundant component of the extracellular matrix (ECM) found in human tissue [27]. Researchers have used 3D culture approaches as a more realistic representation of in vivo conditions. 3D culture helps bridge the gap between in vitro and in vivo models and it is a more accurate representation of the ECM found in the body [27,28].

These parameters were then used to explore mitochondria transfer between ADSCs and HEK293 tumorigenic cells. HEK293 is one of the most common cell lines used due to its low maintenance and robustness. HEK are tumorigenic cells that are used in a variety of research areas, including cell biology and cancer research [29], which makes them an excellent target for proof-of-concept studies. This research will bring us closer to understanding the capabilities of mitochondria transfer and its link to disease, aging, cancer, and the production of energy.

## 2. Results

### 2.1. SCs to SCs Mitochondria Transfer as a Function of Density

**Figure 1:**
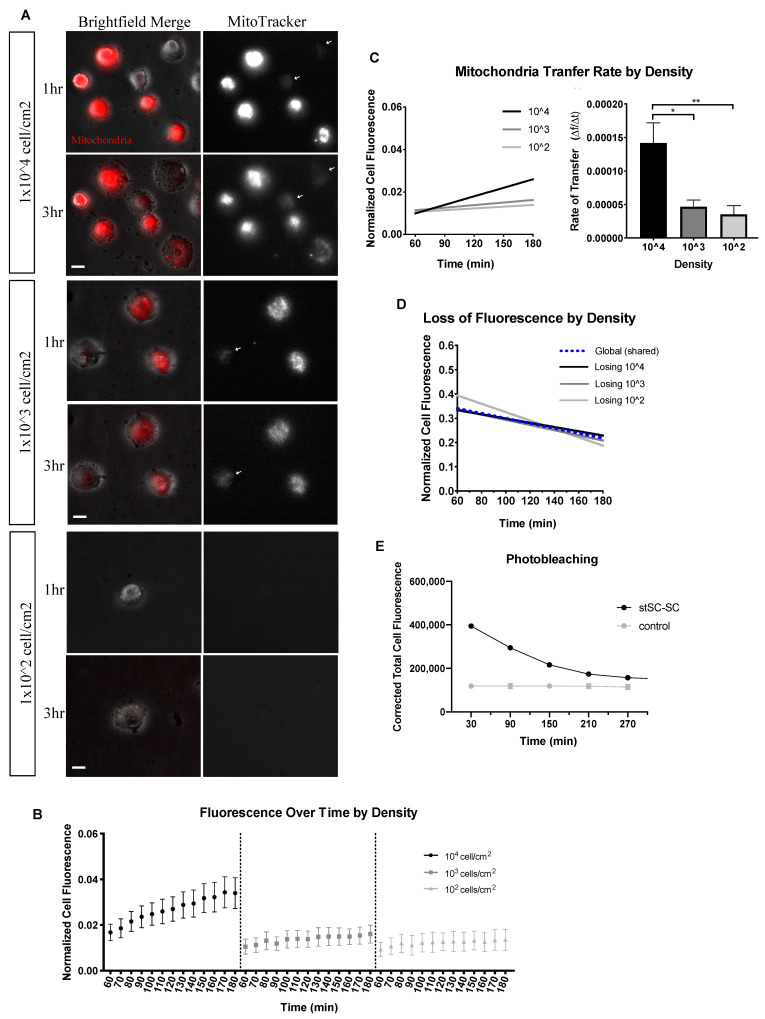
Mitochondria transfer as a function of density. (**A**) Two-hour time-lapse of co-cultured stained ADSC (Donor) and unstained ADSC (Recipient) plated at 10^4^, 10^3^ and 10^2^ cell/cm^2^. Cells were plated on 35 mm 2D culture-treated plastic dishes. Scale bar is 20 μm. Left images are brightfield and MitoTracker merged; right images are MitoTracker only. White arrows indicate recipient cells receiving mitochondria. (**B**) Normalized mean cell fluorescence of recipient cells plotted over time (min) at each density. (**C**) Data were fit to a linear model. Recipient cell transfer rates were computed and displayed by density. Statistical analysis was conducted using one-way ANOVA and Tukey’s multiple comparisons test (10^4^ *n* = 50, 10^3^ *n* = 25 and 10^2^ *n* = 10, over 4 independent experiments). (**D**) Donor SCs were fit to a linear model as displayed. (**E**) Stained SCs cultured with unstained SCs (stSC–SC) versus stained SCs cultured alone to assess photobleaching. Significance is presented as * *p* < 0.05, ** *p* < 0.01.

Mitochondria within cells were labeled with MitoTracker Red to monitor movement. Stained ADSC (donor) and unstained ADSC (recipient) were co-cultured on plastic culture dishes at three densities (Figure 1A)—10^4^, 10^3^ and 10^2^ cells per cm^2^—and incubated for 60 min prior to initiation of experiments. Cell fluorescence was measured every ten minutes for two hours. In the first three hours post seeding, cells begin their spreading phase, where they securely attach to the surface of the dish and begin to flatten and spread. At each density, we looked at the change in fluorescence in recipient cells, which indicates reception of mitochondria. Trends shown in Figure 1B suggest that higher cell densities produce greater mitochondria movement as indicated by cell fluorescence. We fit a linear model to describe the change in fluorescence over time. Corrected total cell fluorescence (CCF) was used as a measure of the mitochondria population within a cell and not an individual mitochondrion. CCF, further described in the methods section of this paper, accounts for background noise and cell size. CCF was used to investigate the movement of mitochondria and calculate the rates of mitochondrial transfer.

Analysis of the recipient cell population generated a significant difference between the transfer rates of 10^4^ cell/cm^2^ and both lower densities. As depicted in Figure 1C, 10^4^ cell/cm^2^ had a transfer rate 3-fold higher than 10^3^ cell/cm^2^ and 4-fold higher than 10^2^ cell/cm^2^. In this SC to SC system, donor cells appeared to decrease in florescence at a similar rate independent of cell density (Figure 1D). To assess the effects of photobleaching on loss of fluorescence, we cultured stained SCs with unstained SCs and compared it to stained SCs cultured alone. Results showed no detectable decrease in fluorescence over time for stained SCs plated alone indicating minimal photobleaching (Figure 1E). Furthermore, our experiments and conclusions highlight the recipient cell increase in florescence rather than the loss of the donor. While this analysis delved into the effects of density on mitochondria transfer, it is important to also consider the proximity between cells. In the body, recipient and donor cells can be found at various distances apart, with some in direct contact and others at a distance. This distance between cells was more pronounced at lower seeding densities and could indicate a preferred mode of mitochondrial transfer. For this reason, we investigated the transfer rates of recipient cells based on their proximity to a donor cell.

**Figure 2:**
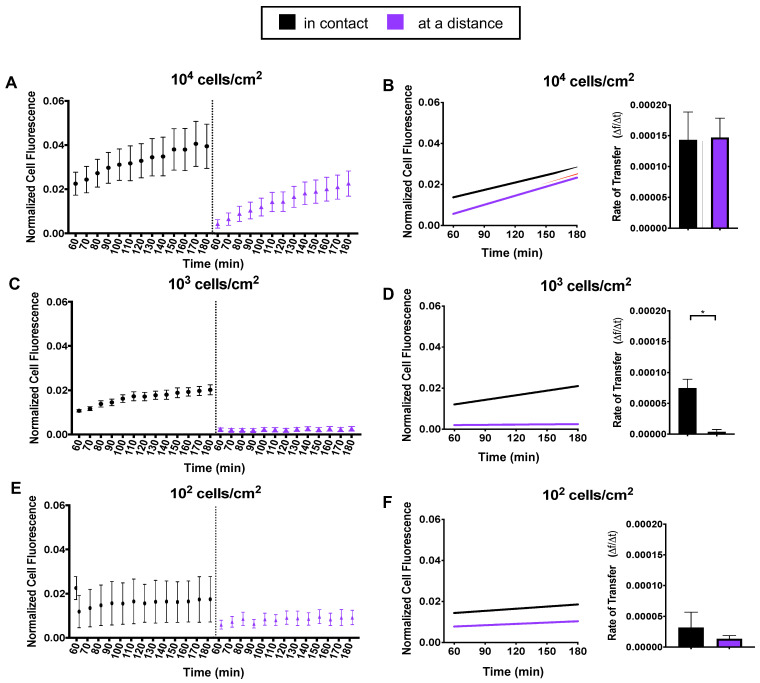
Mitochondria transfer as a function of distance. Recipient cells were divided into two distance groups: in contact or at a distance from a donor cell. (**A**,**C**,**E**) For each density, normalized mean cell fluorescence of recipient cells was plotted by distance groups every 10 min for two hours. (**B**,**D**,**F**) Data were fit to a linear model and recipient cell transfer rates were computed and displayed as a function of distance. Statistical analysis of 10^4^ and 10^3^ was conducted using one-way ANOVA and Tukey’s multiple comparisons test. Statistical analysis of 10^2^ was conducted using an unpaired *t* test (10^4^ *n* = 50, 10^3^ *n* = 25 and 10^2^ *n* = 10, over 4 independent experiments). Statistically significant differences were detected in at 10^3^ density between distance groups. Significance is presented as * *p* < 0.05.

We put recipient cells into two groups: (i) in contact—recipient cells that were in contact with a donor cell or (ii) at a distance—recipient cells that were at least 250 μm away from any donor cell; thus, we expect that recipient cells at a distance could not receive mitochondria by direct contact. Trends seen in Figure 2A,C,E suggested that density and distance coincide. We fit a linear model and compared transfer rates as a function of cell distance for our three density groups. At 10^4^ cells/cm^2^, we observed no difference in mitochondrial transfer rates between cells in contact or cells at a distance (Figure 2B). At 10^3^ cells/cm^2^, we noticed the most remarkable difference between groups (Figure 2D). Cells in contact had a mitochondrial transfer rate 19-fold higher than cells at a distance. At 10^2^ cells/cm^2^, we observed a minor difference, with cells in contact having a mitochondrial transfer rate 1.5-fold higher than cells at a distance (Figure 2F).

### 2.2. Extracellular Matrix Effects on Mitochondria Transfer between SCs

**Figure 3:**
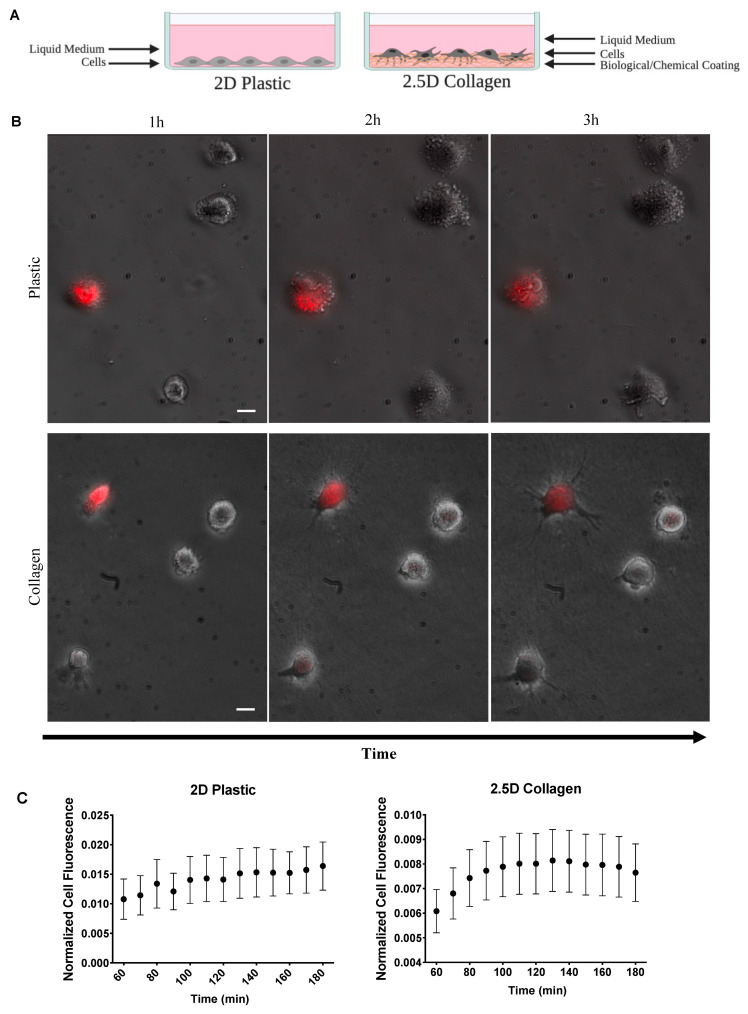
Effects of ECM on mitochondria transfer. (**A**) Illustration of 2D and 2.5D culture dishes. (**B**) Two-hour time-lapse of co-cultured stained ADSCs and unstained ADSC plated on 2D plastic and 2.5D collagen (2D *n* = 25 over 4 independent experiments, 2.5D *n* = 30, over 2 independent experiments). Scale bar is 20 μm. (**C**) Normalized mean cell fluorescence of recipient cells plotted every 10 min for two hours for 2D and 2.5D ECM to visualize trend over time.

We examined SC to SC communication in 2D and 2.5D culture as illustrated in Figure 3A. We used a 2.5D collagen model to represent a physiological environment and a more relevant model to study communication between cells. Cells were stained and experiments were conducted as previously stated. Cell were seeded only at 10^3^ cells/cm^2^ on culture-treated plastic (2D) and collagen (2.5D) dishes (Figure 3B).

**Figure 4:**
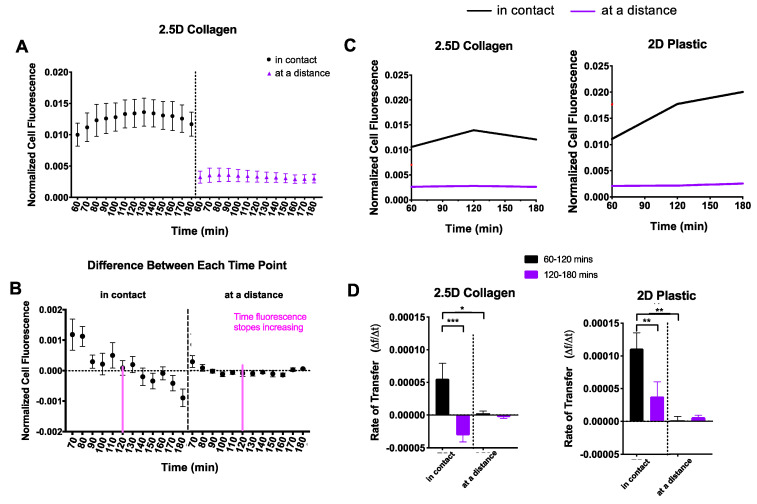
Comparison of 2D and 2.5D culture. Recipient cells were divided into two distance groups based on their distance from donor cells: (i) in contact and (ii) at a distance. (**A**) For each culture system, normalized cell fluorescence of the recipient cell population was plotted based on distance groups every 10 min for two hours. (**B**) The difference in fluorescence between each successive timepoint was calculated to determine peak fluorescence. The dotted line indicates a difference of zero and was the threshold used to identify peak fluorescence. This peak is indicated by a pink line (120 min). (**C**) Data were fit to a segmental linear regression model, intervening at 120 min. (**D**) Mitochondrial transfer rates were computed and displayed as a function of distance. One-way ANOVA and Tukey’s multiple comparisons test were performed. Significance is presented as * *p* < 0.05, ** *p* < 0.01, and *** *p* < 0.001.

We observed a striking trend in recipient cell fluorescence over time in 2.5D culture (Figure 3C). Cell fluorescence appeared to immediately increase before reaching a plateau phase. In comparison, the 2D dishes appeared to steadily increase over time. To explore this further, we divided the cells into the distance groups as previously described: cells in contact and cells at a distance (Figure 4A). Since the 2.5D trend did not appear to increase at a constant linear rate, we split the data into two sections. The first section included all timepoints up to the peak in fluorescence and the second section started at the peak through the remaining timepoints. To determine the peak in fluorescence, we calculated the difference in fluorescence between each successive timepoint (Figure 4B). We identified the timepoint at which fluorescence no longer increased as 120 min, indicated by the pink line in Figure 4B. Using this timepoint, we conducted a segmental linear regression for both 2D and 2.5D groups (Figure 4C).

In 2.5D culture, recipient cells “in contact” displayed an increase in mitochondrial transfer followed by a notable decline into the third hour (Figure 4D, left), whereas recipient cells “at a distance” displayed a mitochondrial transfer rate of nearly zero, the entire duration. In 2D culture, recipient cells “in contact” showed a steep mitochondrial transfer rate that persisted into the third hour but at a significantly slower rate (Figure 4D, right), while recipient cells “at a distance” displayed a mitochondrial transfer rate of nearly zero much like those in 2.5D culture.

**Figure 5:**
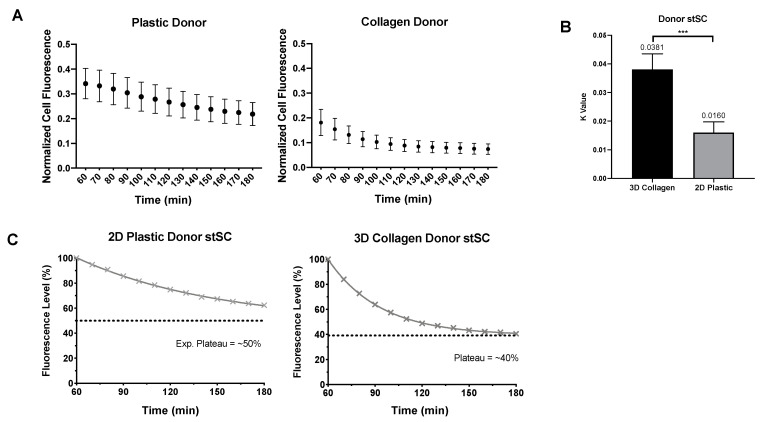
Comparison of donor 2D and 2.5D culture. (**A**) Normalized mean cell fluorescence of donor cells in 2D plastic and 2.5D collagen every 10 min for two hours. (**B**) Each donor cells was fit to an exponential plateau model to obtain k values. Statistical analysis was preformed using a nonparametric Mann–Whitney test. (**C**) Data were transformed to percentage of fluorescence assuming all cell start with 100% of their own mitochondria. Distinct difference in loss of fluorescence can be visualized between 2D plastic and 2.5D collagen culture. Significance is presented as *** *p* < 0.001.

To investigate how the ECM affects the ability of a cell to release mitochondria, we analyzed and compared the reduction in donor cell fluorescence as an indication of mitochondrial transfer (Figure 5A). We fit an exponential decay model to both the 2D and 2.5D datasets. The exponential decay model is defined by the following equations: F(t) = (F0 − Plateau)e^−kt^ + Plateau), where F is the fluorescence value, F0 is the maximum value, Plateau is a constant that indicates the minimum value, t is time and k is referred to as the rate constant. For this model, the rate constant (k) indicates the rate of mitochondrial transfer.

In 2.5D culture, donor cells produced a higher rate of mitochondrial transfer (k value of 0.0381) than the donor cells in 2D culture (k value of 0.0160) (Figure 5B). Furthermore, donor cells in 2.5D culture not only reached their plateau faster but they also plateau lower than donor cells in 2D culture (Figure 5C). Together, our analysis showed that 2D recipient cells increase more rapidly in fluorescence (presumptive mitochondrial uptake) than the 2.5D recipient cells. However, the donor cells in 2.5D culture showed a more rapid depletion of fluorescence (presumptive mitochondrial donation) and lost more fluorescence overall than donor cells in 2D culture.

### 2.3. Mitochondria Sharing between HEK293 Tumorigenic Cells and Human Adipose-Derived Stem Cells

We utilized Rho0 SCs as a model for cells under stress with limited oxidative phosphorylation capabilities and HEK293 as a model for cancer cells. Healthy SCs were stained and co-cultured in the following three groups of cells: stained SCs with unstained SCs (stSC–SC), stained SCs with unstained HEK293 tumorigenic cells (stSC–HEK) and stained SCs with Rho0 SCs (stSC–Rho0). stSC–SC culture group acted as our control, stSC–Rho0 acted as our stress group and stSC–HEK as our tumorigenic group. Culture groups were incubated for 30 min prior to initiation of experiments. Cell fluorescence was measured every 30 min for 24 h. Stained cells were considered donor cells and unstained cells were considered recipient cells.

**Figure 6:**
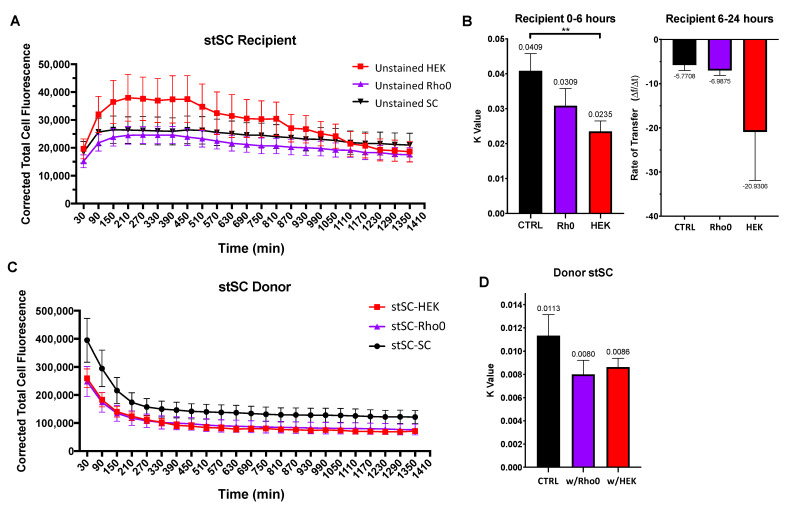
Effect of tumorigenic cells on SC mitochondria transfer. Cells were cultured in three culture groups (i) stSC–SC (ii) stSC–HEK and (ii) stSC–Rho0. (**A**) Mean cell fluorescence of recipient cells plotted every 60 min over 24 h. (**B**) Recipient cell data were split into two periods: (i) 0–6 h and (ii) 6–24 h. For 0–6 h, each cell was fit to an exponential plateau model with a robust regression fit to compute k values; and for 6–24 h, to a robust linear regression to compute transfer rates. Statistical analysis was preformed using the Kruskal–Wallis test to compare to the control group. (SC *n* = 19, HEK *n* = 23, Rho0 *n* = 20, over 3 independent experiments.) (**C**) Mean cell fluorescence of donor cells plotted every 60 min over 24 h. (**D**) Each donor cell data were fit to an exponential decay model with a robust regression fit and k values were computed. Statistical analysis was preformed using the Kruskal–Wallis test to compare to the control group. (stSC w/SC *n* = 23, stSC w/HEK *n* = 20, stSC w/Rho0 n = 19, over 3 independent experiments.) Significance is presented as ** *p* < 0.01.

Data revealed recipient cell populations rapidly increasing fluorescence before stabilizing over time (Figure 6A). Notably, HEK recipient cells appeared to accept 2-fold more donor cell mitochondria than Rho0 SCs and control SCs, which was striking given their typically smaller cell size. Additionally, recipient HEK cells appear to decrease in fluorescence after plateauing, suggesting a loss or degradation of mitochondria (Figure 6A, red line). Data were split into two sections for calculation and analysis of mitochondrial transfer rates. The first section included the immediate increase in fluorescence until plateau which we identified to be 0–6 h. The second section included the remaining 6–24 h. Based on trends of the data, the first section was analyzed using an exponential plateau model for the recipient cell mitochondrial transfer. The exponential plateau fit is defined by the following equation F(t) = A(1 − e^−kt^), where F is the fluorescence value, A is a constant that indicates the maximum plateau value, t is time and k is the rate constant. In this model, the rate constant (k) is proportional to the time it takes to reach half the total amount of mitochondria accepted and was used to identify the rate of mitochondrial transfer, a smaller k value signifies a faster mitochondrial transfer. The second section was fit to a linear model, where the slope indicates the rate of mitochondrial transfer.

HEK recipient cells displayed a faster mitochondrial transfer rate (k = 0.0235) than that of both Rho0 SCs (k = 0.0309) and control SCs (k = 0.0409) (Figure 6B, left), whereas Rho0 recipient cells displayed a mitochondrial transfer rate similar to that of the control SCs. Together, we concluded that that HEK recipient cells can accept higher quantities of donor mitochondria and at a faster rate than both control SCs and Rho0 SCs. After plateauing, fluorescence decreased for all culture groups. HEK recipient cells appeared to lose fluorescence faster (slope = −20.9306); however, there was no statistical difference detected in the rate compared with that of the control SCs (slope = −5.7708) or Rho0 SCs (slope = −6.9875) (Figure 6B, right).

Next, we investigated the transfer rates of donor SCs to determine whether they were affected by the cell types neighboring them. We fit an exponential decay model as described earlier to obtain k values as a measure of mitochondrial transfer rates (Figure 6C). We found no statistical difference between k values of donor SCs culture with SCs, Rho0 cells or HEK cells (Figure 6D). This suggests that the ability of donor SCs to transfer mitochondria is not drastically influenced by the cell types that surround them. Moreover, the ability of recipient cells to accept mitochondria appears to be cell type specific.

**Figure 7:**
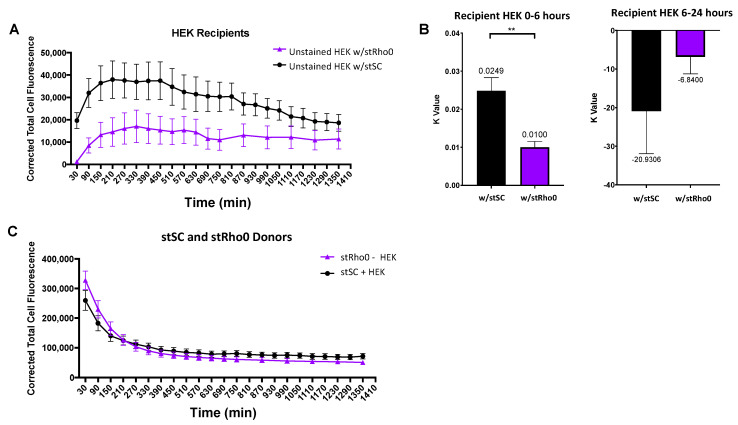
Analysis of mitochondria transfer between (i) stSC–HEK and (ii) stRho0-HEK. (**A**) Mean cell fluorescence of recipient cells plotted every 60 min over 24 h. (**B**) Recipient cell data were split into two periods: (i) 0–6 h and (ii) 6–24 h. For 0–6 h, each cell was fit to an exponential model with a robust regression fit to compute k values. For 6–24 h, each cell was fit to a robust linear regression to compute transfer rates. Statistical analysis was preformed using a nonparametric Mann–Whitney test (HEK w/stSC *n* = 23, HEK w/stRho0 *n* = 20, over 3 independent experiments). (**C**) Mean cell fluorescence of donor cells plotted every 60 min over 24 h. Significance is presented as ** *p* < 0.01.

To investigate the ability of HEK cells to selectively accept mitochondria from healthy cells, we co-cultured two groups: stained Rho0 SCs co-cultured with unstained HEK cells (stRho0-HEK) and stained healthy SCs co-cultured with unstained HEK cells (stSC–HEK). Identified cell types were stained and plated and culture groups were incubated for 30 min prior to initiation of experiment. Trends for both culture groups displayed an increase in fluorescence before plateauing and decreasing over time (Figure 7A). Strikingly, HEK recipient cells appear to accept over 3-fold more mitochondria from healthy SCs than they did from Rho0 SCs. Data were split into two sections and analyzed as previously stated, 0–6 h and 6–24 h. HEK recipient cells cultured with healthy SCs had a significantly higher mitochondrial transfer rate (k = 0.0249) than those cultured with Rho0 SC (k = 0.0100) (Figure 7B, left). After plateauing, HEK recipient cells cultured with healthy SCs decrease in fluorescence over 4-fold faster than those cultured with Rho0 SCs (Figure 7B, right); however, no statistical difference was seen. Analysis of donor cells revealed no difference in mitochondrial transfer rates between culture groups (Figure 7C).

### 2.4. HEK293 Tumorigenic Cell Migration When Co-Cultured with SCs

We used Image J Fuji Software and tracked HEK movement in three culture groups: (1) HEK alone, (2) HEK co-cultured with healthy SCs and (3) HEK co-cultured with Rho0 SCs. To understand changes in directional migration, we used mean squared displacement over time as our metric [30]. Mean squared displacement (MSD) is defined as: $\mathrm{MSD}(t)>=<{\left(x_{i}\left(t_{0}+t\right)-x_{i}\left(t_{0}\right)\right)}^{2}+{\left(y_{i}\left(t_{0}+t\right)-y_{i}\left(t_{0}\right)\right)}^{2}>$, where *x_i_* and *y_i_* denote the position of the ith cell in a laboratory frame and *t* is the time. When MSD is plotted over time, the data can be fitted into a model expressed by the equation MSD = *t*^α^. In this equation, *t* is time and α is an exponential constant that can be used to differentiate between different types of migration. When α is equal to 1, the movement is said to be random. When α is higher than 1 the movement is super diffusive, in other words, it is highly directional.

**Figure 8:**
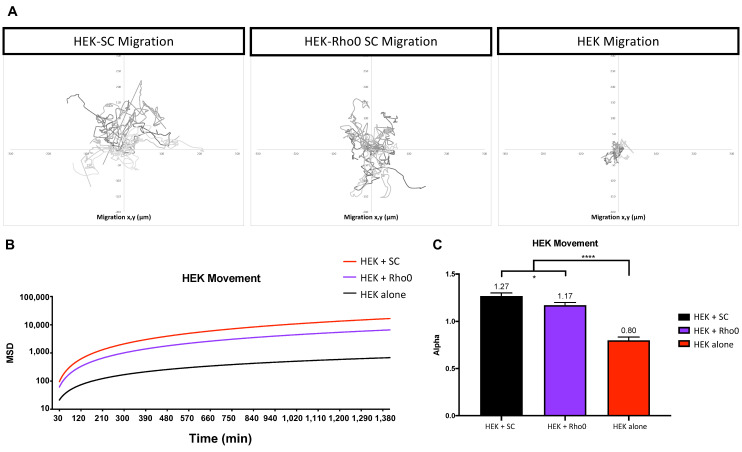
HEK293 cell migration dependence on external cues. HEK movement was analyzed in three groups (i) HEK cultured with SCs, (ii) HEK cultured with Rho0 SCs and (iii) HEK cultured alone. Culture groups were seeded at a density of 10^3^ cells/cm^2^. (**A**) Graphical representation of HEK migration over 24 h using x and y coordinates. (**B**) MSD fit to a model defined as MSD = t^α, where α is a constant to distinguish between types of movement. (**C**) Alpha values of each culture group for visualization. Statistical analysis was preformed using one-way ANOVA and multiple comparison (*n* = 27 for each group, over 2 independent experiments experiments). Significance is presented as * *p* < 0.05, **** *p* < 0.0001.

Migration maps suggested increased movement when HEK were cultured with healthy SCs or Rho0 SCs (Figure 8A). Our results indicated that, alone, HEK exhibit random movement (α = 0.80). However, HEK co-cultured with healthy SCs or Rho0 SCs exhibit highly directional movement (α = 1.27, 1.17, respectively) (Figure 8B,C). These results suggest that HEK may be responding to an external cue released by SCs. In contrast, HEKs cultured alone produced movement resembling random motion, which would be expected in the absence of any external cues.

## 3. Discussion

To better understand the ability of stem cells to share mitochondria with tumorigenic cells, we first determined the ideal parameters to observe mitochondrial transfer from SCs. Mitochondrial transfer can occur through direct contact or extracellular vesicles. Previous research [25] showed that lower seeding densities produce a larger amount of EVs and suggest that mitochondrial transfer via EVs is higher at 10^2^ cells/cm^2^. We did not analyze the production of EVs to confirm this increase at lower densities; however, our study showed that higher seeding densities produced higher rates of mitochondrial transfer overall. When using EVs to transfer material between cells, higher production does not equal higher mitochondrial transfer rates if the donor and recipient cells are not close enough. Additionally, production of EVs might be elevated at lower densities [25], but there is no guarantee that these EVs contain mitochondria. In fact, EVs are likely to contain mRNA or proteins [31,32]. To understand this, we concentrated our efforts on determining the parameters that influence mitochondrial transfer over time, focusing on the distance between donor and recipient cells rather than the mode of transport itself.

Based on the distance between recipient and donor cells, we identified two groups: (i) in contact—recipient cells in contact with a donor cell and (ii) at a distance—recipient cells at a distance of at least 250 μm from any donor cell. We assume that cells in contact can receive mitochondria through direct methods or via extracellular release from donors, but cells at a distance can only receive mitochondria via extracellular release from donors. In a previous study [33], researchers found that PC12 cells transferred mitochondria to stressed PC12 cells via nanotubes formed within a distance less than 9–16 μm; thus, it is reasonable to assume that stem cells at a distance above 250 μm would not have nanotubes formed within the timeframe of our observations.

At a seeding density of 10^4^ cells/cm2, most cells were touching or very close to one another. Results showed no differences in mitochondrial transfer between cells in contact or at a distance. This could indicate that at high densities, the medium is flooded with EVs containing mitochondria, resulting in the distance being inconsequential. In contrast, at 10^2^ cells/cm^2^, most cells were far away from one another (250 μm group) and mitochondrial transfer rates were significantly decreased. Even when a recipient cell was close to a donor cell, transfer rates were still significantly lower than those of higher densities. This could indicate that the availability of transferable mitochondria is too low or the cell–cell communication for preferred mitochondrial transfer is limited, such as the formation of nanotubes over EVs. Therefore, distance between the donor and recipient cell must be considered as this may indicate a preferred or optimal mode of mitochondrial transfer. Seeding cells at 10^3^ cells/cm^2^ provided a statistical difference in transfer rates based on the distance between recipient and donor cell. This density provided the ideal conditions to analyze mitochondrial transfer rates as a function of distance between cells and was used for our subsequent experiments.

We investigated mitochondria transfer on 2.5D collagen to represent a more physiological environment. The 2.5D assay facilitates imaging while still changing the substrate to a more physiologically relevant matrix (in our case, collagen). For cells in contact, we observed higher transfer rates in 2D than 2.5D, as shown in Figure 4D. This could be explained by multiple phenomena. 2D culture has been seen to cause stress on a cell [26,34] and could promote a stress response in cells, resulting in more mitochondria being released. This could explain the steep and constant increases in the mitochondrial transfer rate in a 2D culture system. Additionally, cells in 2.5D culture are embedded in a collagen matrix, which could result in the recipient cells losing access to the free-floating EVs with mitochondria. While the calculations of the EV movement in a dense collagen matrix are more complex than simple diffusion and outside the scope of this project, it is safe to assume that their movement is hindered further than in 2D, where there is no extracellular matrix. In clinical applications, it is important to consider how far a cell or EV must travel and through what environment. This could affect how much and how often a treatment should be administered for maximum effect.

Previous research has shown that cancer cells may release signals that trigger other cells to help them thrive [35], including stem cells [36]. Moreover, injured cells under oxidative stress have been reported to activate mitochondria transfer in SCs [11,12]. This led us to investigate whether tumorigenic cells mimic the signaling response of injured or stressed cells in order to receive mitochondria from SCs. Most notably seen in Figure 6A, HEK tumorigenic cells received more mitochondria from donor SCs than did control SC. This suggests that HEK need more mitochondria and have the ability to accept more mitochondria from the environment. Thus, the ability of the recipient cell to accept foreign mitochondria may play a bigger role than the ability of the donor cell to donate. In fact, a large area of academic and industry research has been looking at the content of stem cell extracellular vesicles to understand what makes them better at being internalized than other drug delivery vehicles [37,38,39,40,41,42]. Future research to elucidate the mechanisms responsible for mitochondria transfer from stem cell to tumorigenic cells could uncover ways to manipulate drug delivery vehicles to enhance internalization to a recipient cell [43,44,45,46].

It is known that migration plays a key role in homeostasis and wound healing but it is also a vital process in disease, specifically in cancer [47,48]. Abnormal cell migration plays a role in cancer metastasis [48] and is often the target of many therapeutic drugs [49,50]. Our results suggest that HEK cells are sensitive to the presence of ADSCs, presumably due to the expression of chemotactic cues, as indicated by the increased directional migration (Figure 8). The chemotactic relationships between cancer cells and stem cells have been observed previously [51]. ADSCs produce stromal cell-derived factor 1 (SDF-1) [52], and tumor growth and HEK specifically have shown sensitivity to SDF-1 [53,54]. Thus, this could be responsible for the observed increase in directional migration. In future, experiments could identify and analyze the chemotactic molecules produced by ADSCs in the presence of HEK.

In patients, adipocytes have been associated with the tumor microenvironment and been shown to contribute to tumor growth by functioning as an energy source [55]. Additionally, within the tumor environment, MSC have exhibited the ability to release a number of factors which play a role in enhancing cancer stem cell properties and migration abilities [56,57]. MSC cells have been seen to localize with breast carcinomas [58,59], and promote metastasis [58,60]. Conversely, mitochondrial transfer is not exclusively exploited by cancerous cells. This mechanism has been observed between various cell types and is considered to be a pertinent ability for SCs to rescue dying or damaged tissue [61]. In response to injury or inflammation, SCs have been known to donate various molecules including mitochondria to rescue cells from apoptosis [33,61]. The donation of mitochondria plays a key role in the modulation of ROS and maintenance of homeostasis in tissue [62]. Knowing that SCs have opposing effects on tissue maintenance and growth is vital to thoroughly evaluate and investigate this duality in clinical and/or therapeutic applications involving SCs. Furthermore, we believe there is an opportunity to identify and harness the ability to manipulate cancer cells through their need for energy. This approach could give rise to pivotal therapies for cancer treatment. For example, cancer cells could be strategically attracted to areas in which they do not present a lethal effect and/or to be more easily extracted. Moreover, the mitochondrial transfer pathway could be used as a trojan horse to internalize therapeutic products into invasive tumors.

## 4. Materials and Methods

### 4.1. Cell Culture and Maintenance of Cell Lines

Human adipose-derived stem cells (ADSC) were obtained from Thermo Fisher, Waltham, MA, USA and are extracted from human lipoaspirate tissue of a single donor, expanded for one passage and cryopreserved. HEK923 tumorigenic cells were obtained from American Type Collect (ATCC, Manassas, VA, USA) and are derived from embryonic kidney tissue of humans and cryopreserved. HEK293 are a robust rapidly growing cell line commonly used for cancer research. ADSC were thawed and cultured in MesenPRO Reduced Serum (ThermoFisher, catalog no. 12746012, Waltham, MA, USA) growth medium for 24 h and then switched to a standard medium. The standard medium consisted of Dulbecco’s Modified Eagle Medium, high glucose, GlutaMAX™ Supplement (Gibco, ThermoFisher, catalog no. 10566016, Waltham, MA, USA), 10% Fetal Bovine Serum (FBS) and 1% Penicillin-Streptomycin antibiotic (Gibco, ThermoFisher catalog no. 15070063, Waltham, MA, USA). HEK293 cells were thawed and cultured in the standard medium from day one. All cell lines were thawed and incubated at 37 °C and 5% CO_2_ for a minimum of 24 h prior to initiation of experiments. All experiments were cultured in the standard culture medium and the medium was changed every 2 days. Cells with stained mitochondria are referred to as donor cells and unstained cells are referred to as recipient cells. Cells were seeded on 35 mm dishes at various densities; 10^4^, 10^3^, and 10^2^ cells per cm^2^. For all co-culture experiments, the seeding density was split 50/50 donor cells/recipient cell. Cells were counted and mixed in a tube and plated on a dish. All SC–HEK co-culture experiments were performed at a seeding density of 10^3^ cell/cm^2^, which was split 50/50 SC/HEK. The abbreviation SC was used in place of ADSC for adipose-derived stem cells in certain Figures to save space.

### 4.2. 2.5D Cell Matrix Preparation

Our semi two-dimensional 2.5D culture model incorporates a thick layer of collagen to simulate the ECM structure found in the human body. In contract to a traditional 2D system, this layer of collagen provides cells with an ECM more physiologically relevant to tissue in the human body. As depicted in Figure 3A, this layer affects the ability of a cell to attach and form protrusions, which allows for a more realistic representation of cell attachment and nanotube formation. The collagen matrix was formed using PureCol and TeloCol-3, Type I Collagen Solution, 3 mg/mL (Bovine) (Advanced BioMatrix, catalog no. 5005, 5026, Carlsbad, CA, USA) and prepared according to the manufacturer’s recommendations. In a sterile environment, collagen was prepped and poured in 35 mm dishes at a depth of 500 μm and incubated at 37 °C and 5% CO_2_ until solidified.

### 4.3. Generation of Rho0 Cell Line

Rho0 cells were prepared as previously published [63] to decrease their mtDNA levels. Cells were cultured with ethidium bromide (EtBr) at a concentration of 100 nM for 2 weeks prior to use in experiments. EtBr was supplemented in each media change.

### 4.4. Quantification and Visualization of Mitochondria

Fluorescent staining of mitochondria was performed using MitoTracker Red (Thermo Fisher, catalog no. M22425, abs/em ~581/644 nm, Waltham, MA, USA), a fluorescent dye that accumulates in the mitochondrial matrix of live cells and binds to free thiol groups on mitochondrial proteins. Staining of cells was performed by adding 250 nM of MitoTracker Red into the culture mdium for 20 min at 37 °C and 5% CO_2_. Cells were washed a minimum of three times with PBS, detached with 0.05% Trypsin (Thermo Fisher, catalog no. 25300054, Waltham, MA, USA) for 5–10 min and counted for seeding experiments. Donor cells and recipient cells were grown on separate culture dishes. Donor cells were stained, washed, and detached for counting. Recipient cells were washed, detached, and counted. Donor cells and recipient cells of specified concentrations were then mixed in a tube and transferred to a culture dish. Cell were kept at 37 °C and imaged at specific time points over a set length of time as described in each figure. Time-lapses were obtained using the TE200U Nikon Inverted Fluorescence Microscope at a 20× objective. Culture dishes were kept at 37 °C inside the microscope and time-lapse images were taken at 250 ms exposure with Thorlabs 3000 K, 2000 mW (Min) Mounted LED, 700 mA and Thorlabs T-Cube™ LED Driver (Thorlabs, Newton, NJ, USA) set at a maximum of 1.2 Amperes. Using Fiji ImageJ software, cells were traced manually to measure cell size, cell mean fluorescence, and background mean fluorescence. Microsoft Excel was used to calculate the corrected total cell fluorescence (CCF). CCF accounts for background noise and cell size. CCF is the (mean cell fluorescence × cell size) = (cell size × background mean fluorescence). CCF was normalized on a scale of 0–1, 0 being the no fluorescence readings and 1 being the brightest. Values were normalized per experiment using the equation: N(F(t)) = (F(t))/(F(MAX)), where N is the normalized CCF, F(t) is the CCF at a given time and F(MAX) is the max CCF for the experiment.

### 4.5. Statistical Analysis

All calculations were performed in Microsoft Excel. Appropriate statistical analyses were performed and curves were fit using GraphPad Prism v8. Results are reported as the mean and standard error of the mean (SEM) and p values less than 0.05 were considered significant. Statistical analysis was performed using Prism v8, and statistical analysis was conducted as stated in each figure. Significance is presented as * *p* < 0.05, ** *p* < 0.01, *** *p* < 0.001, and **** *p* < 0.0001.

## 5. Conclusions

In this study, we sought to unveil the parameters that affect adipose-derived stem cell mitochondria transfer. We found that density and cell to cell distance played a role in mitochondria sharing. We also found that the time dynamics of mitochondria sharing were different in 2D plastic vs. 2.5D collagen microenvironments. We found that stem cells can transfer mitochondria to HEK293 tumorigenic cells and that their co-culture induces a migratory response. Understanding the mechanism behind the ability of tumorigenic cells to sense and receive resources from their microenvironment could enhance their growth, including signals and resources from stem cells, and could lead to interesting therapies that reduce the risk of tumor metastasis during stem cell treatments.

## Data Availability

Not applicable.
